# Editorial: The role of paternal obesity on offspring health

**DOI:** 10.3389/fendo.2024.1428886

**Published:** 2024-06-11

**Authors:** Xiaoxu Xie

**Affiliations:** ^1^ Clinical Research Unit, The Second Affiliated Hospital, Fujian Medical University, Quanzhou, China; ^2^ Department of Epidemiology and Health Statistics, School of Public Health, Fujian Medical University, Fuzhou, China

**Keywords:** obesity, fathers, parental obesity, offspring health, paternal effects, male fertility

In recent years, obesity has emerged as a major global public health challenge, which not only affects the physical health of individuals, but also has far-reaching socioeconomic implications. Maternal obesity has a direct impact on fetal and infant development through nutritional and metabolic status during pregnancy and breastfeeding, while paternal obesity impacts the offspring through factors such as genetics and shared living environment. In studies on the genetic and environmental factors of obesity, the relationship of maternal obesity with offspring outcomes has been widely explored, but the effect of paternal obesity on children’s subsequent outcomes has not been sufficiently emphasized. This Research Topic deeply explores the associations of paternal obesity with offspring health, aiming to provide new insights and recommendations for obesity prevention and control.

As a chronic metabolic disease caused by multiple factors, obesity has a complex pathogenesis involving genetic, environmental, dietary and exercise factors. Among them, genetic factors play an important role in the occurrence of obesity. As an important source of genes for the offspring, the paternal obesity status may profoundly affect the health of the offspring. In addition, environmental factors such as the father’s dietary habits and lifestyle may also affect the progeny health through genetic or epigenetic approaches (1).

High body mass index (BMI) in males is associated with alterations in semen quality, and these alterations may make natural conception difficult. Mele et al. explored the role of antioxidants in regulating the obesity-dependent relationship between circular RNAs (circRNAs) and sperm quality. They demonstrated that antioxidants positively modulate the obesity-dependent circRNA-sperm quality-function axis. By restoring the circRNA profile in sperm, the antioxidants were able to improve sperm viability and motility, potentially enhancing male fertility in obese individuals. Shen et al. investigated the influence of maternal BMI on the value of serum progesterone in predicting clinical pregnancy outcomes in *in vitro* fertilization/intracytoplasmic sperm injection (IVF/ICSI) cycles. The study found a significant interaction between BMI and progesterone concentration on the day of hCG injection to induce ovulation in determining clinical pregnancy rates. These studies highlight the fact that parental obesity can lead to difficulties in natural conception and even poor results with assisted reproductive technologies.

Paternal obesity has been shown to affect a range of reproductive and offspring outcomes including maternal pregnancy complications, fetal growth and development, birth outcomes, and adolescent health (**Figure 1**). Lin et al. assessed the relationship of paternal obesity with fetal growth as well as gestational complications. They found that the risk of preeclampsia, cesarean section, fetal macrosomia, small for gestational age (SGA), and postpartum hemorrhage was significantly higher in the group with obese fathers compared to the normal BMI group. An increasing trend was observed in fetal ultrasonographically measured parameters, neonatal weight, and placental weight with rising paternal BMI, though this difference waned in the obese group. Further analysis indicated the influence of paternal obesity on macrosomia and SGA was modified by maternal obesity. Lin et al. assessed the impact of paternal obesity on maternal and infant health and adolescent prognosis in the long term. The results showed that paternal overweight and obesity were associated with higher risk of gestational hypertension, pregnancy weight gain above recommended guideline values, cesarean section, and macrosomia. Furthermore, the study demonstrated that adolescents born to obese fathers had an increased risk of asthma, anemia, dental caries, hand-foot-and-mouth disease, and obesity.

**Figure 1:**
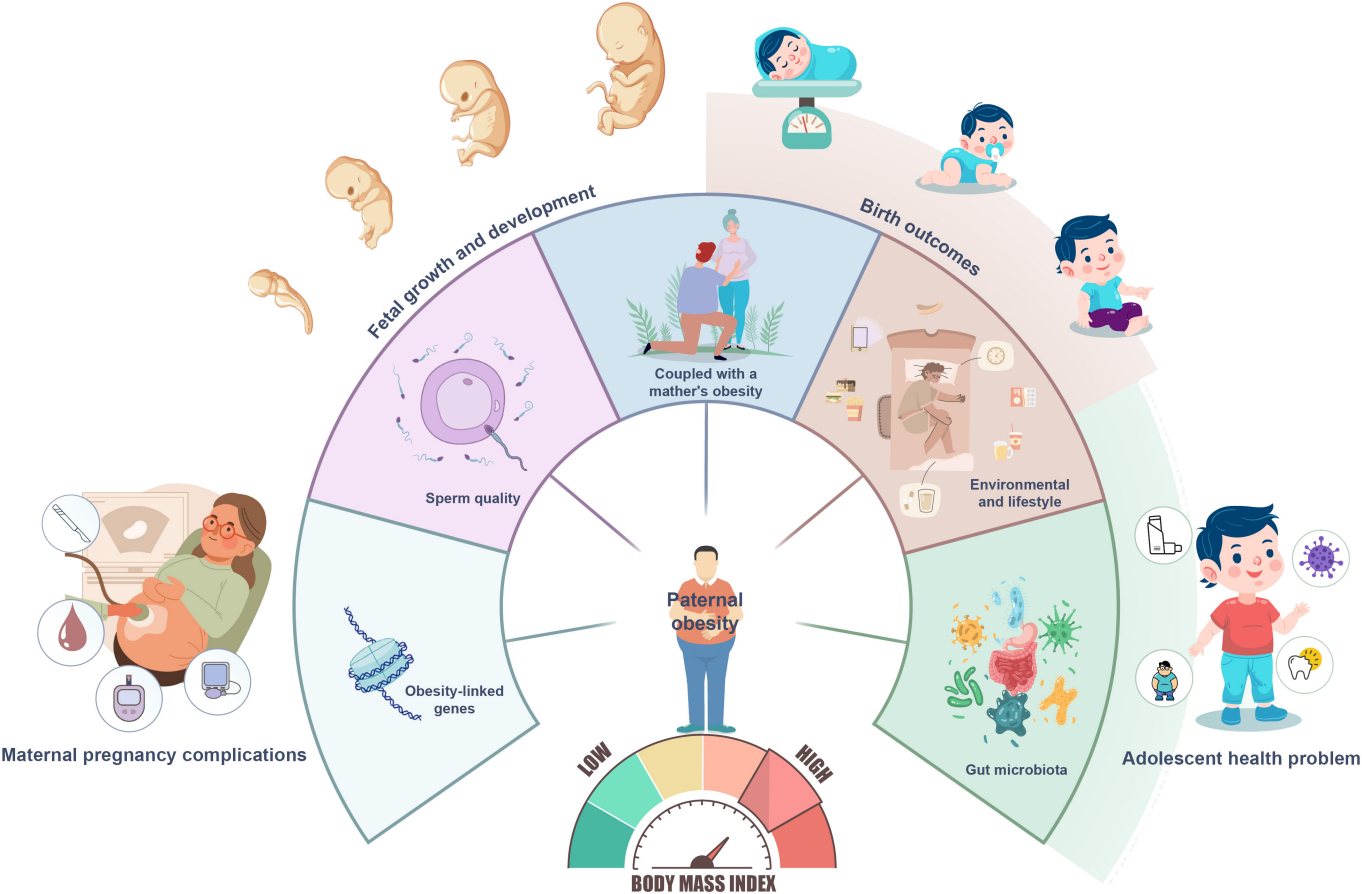
Effects of paternal obesity on offspring health and potential mechanisms.

The potential mechanisms of how paternal obesity adversely affects offspring health are intricate and multifaceted (**Figure 1**). These mechanisms primarily encompass genetic factors, where obesity-linked genes are transmitted to offspring, influencing their energy metabolism, appetite regulation, and fat storage. Epigenetic changes, hormonal imbalances, and elevated scrotal temperature associated with obesity can all negatively impact sperm quality, potentially leading to embryonic abnormalities. Furthermore, maternal obesity, particularly when coupled with a father’s obesity, intensifies the negative impact on fetal development, due to shared unhealthy lifestyle and eating habits. Environmental and lifestyle factors, such as inadequate exercise, unhealthy dietary patterns, and insufficient sleep can be transmitted within families, adversely affecting the health of future generations (2, 3). Recent research has also found that environmental factors that disrupt the gut microbiota can trigger substantial reproductive responses in prospective fathers (4). This suggests the existence of a gut-genital regulatory axis that, when disrupted, may increase the risk of disease in the offspring by affecting placental function. The gut microbiota is a key interface for various environmental factors (e.g., antibiotics or diet) that directly or indirectly affect male germ cells and ultimately the offspring.

It’s crucial to recognize that these mechanisms are interconnected and may interplay to collectively impact offspring health. Notably, weight loss has been shown to reverse the detrimental effects of paternal obesity on fertility. Therefore, proactive measures such as weight management, improved lifestyle and dietary habits, and gut microbiota health should be prioritized by both parents. Physicians should provide tailored advice and support to assist families in achieving these goals.

In conclusion, paternal obesity has significant effects on offspring health, encompassing transgenerational impacts, molecular interactions, and epigenetic changes. The articles in this Research Topic highlight the influence of paternal obesity on fertility, pregnancy outcomes, and progeny health, yet further studies with longer follow-up times, larger offspring samples, and combining multi-omics techniques are needed to elucidate the intricate relations and potential pathways involved. The interaction of maternal and paternal obesity on the health effects of offspring also warrants future study. Filling the many gaps in our understanding of this topic will help develop targeted interventions to improve family planning and offspring health.

## Author contributions

XX: Conceptualization, Writing – original draft, Writing – review & editing.
